# Pertussis Toxin, an Inhibitor of G_αi_ PCR, Inhibits Bile Acid- and Cytokine-Induced Apoptosis in Primary Rat Hepatocytes

**DOI:** 10.1371/journal.pone.0043156

**Published:** 2012-08-10

**Authors:** Golnar Karimian, Manon Buist-Homan, Klaas Nico Faber, Han Moshage

**Affiliations:** Department of Gastroenterology and Hepatology, University of Groningen, University Medical Center Groningen, Groningen, The Netherlands; University of Birmingham, United Kingdom

## Abstract

Excessive hepatocyte apoptosis is a common event in acute and chronic liver diseases leading to loss of functional liver tissue. Approaches to prevent apoptosis have therefore high potential for the treatment of liver disease. G-protein coupled receptors (GPCR) play crucial roles in cell fate (proliferation, cell death) and act through heterotrimeric G-proteins. G_αi_PCRs have been shown to regulate lipoapoptosis in hepatocytes, but their role in inflammation- or bile acid-induced apoptosis is unknown. Here, we analyzed the effect of inhibiting G_αi_PCR function, using pertussis toxin (PT), on bile acid- and cytokine-induced apoptosis in hepatocytes. Primary rat hepatocytes, HepG2-rNtcp cells (human hepatocellular carcinoma cells) or H-4-II-E cells (rat hepatoma cells) were exposed to glycochenodeoxycholic acid (GCDCA) or tumor necrosis factor-α (TNFα)/actinomycin D (ActD). PT (50–200 nmol/L) was added 30 minutes prior to the apoptotic stimulus. Apoptosis (caspase-3 activity, acridine orange staining) and necrosis (sytox green staining) were assessed. PT significantly reduced GCDCA- and TNFα/ActD-induced apoptosis in rat hepatocytes (−60%, p<0.05) in a dose-dependent manner (with no shift to necrosis), but not in HepG2-rNtcp cells or rat H-4-II-E cells. The protective effect of pertussis toxin was independent of the activation of selected cell survival signal transduction pathways, including ERK, p38 MAPK, PI3K and PKC pathways, as specific protein kinase inhibitors did not reverse the protective effects of pertussis toxin in GCDCA-exposed hepatocytes. **Conclusion:** Pertussis toxin, an inhibitor of G_αi_PCRs, protects hepatocytes, but not hepatocellular carcinoma cells, against bile acid- and cytokine-induced apoptosis and has therapeutic potential as primary hepatoprotective drug, as well as adjuvant in anti-cancer therapy.

## Introduction

In chronic and acute liver diseases, the liver is exposed to increased levels of cytokines, reactive oxygen species and bile acids, all of which independently can lead to loss of functional liver mass due to hepatocyte cell death. Concomitantly, hepatic stellate cells become activated, start proliferating and produce excessive amounts of extracellular matrix proteins leading to liver fibrosis, which may progress to end-stage liver disease [1]. Hepatocyte cell death can occur via apoptosis, necrosis or a combination of these different types of cell death [2]. Apoptosis is an energy-dependent process, resulting in the formation of apoptotic bodies. Apoptotic bodies are cleared by surrounding phagocytizing cells that minimize inflammation. In contrast, uncontrolled apoptosis and (secondary) necrosis trigger inflammation in the liver [3], [4]. Despite worldwide efforts to establish therapeutic strategies for liver injury, end-stage liver disease remains a high burden for public health due to the lack of effective treatments. Excessive hepatocyte apoptosis is often observed in liver disease and, as this is a highly controlled cellular mechanism, drugs and therapeutic strategies to prevent hepatocyte apoptosis may help to maintain sufficient liver mass and function [1].

Recently, G-protein coupled receptors (GPCRs) have been suggested as new drug targets to treat cardiac diseases and cancer, as GPCRs play crucial roles in the regulation of cell proliferation, angiogenesis, cell survival and apoptosis [5], [6]. GPCRs are the largest family of membrane proteins and are essential nodes of communication between the internal and external environment of the cells. Over 300 GPCRs have been reported in human and rodents [7]. Upon activation by agonists, GPCRs activate heterotrimeric G-proteins (G_α_βγ). These subunits subsequently activate second messengers (e.g. cAMP, Ca^2+^ and protein kinases), submitting the GPCR induced-signal to the intracellular targets. Heterotrimeric G-proteins are divided into 4 families (i.e., Gαs, Gαi, Gαq/11 and Gα12/13) based on the G_α_ subunit sequence identity and signaling activity [8].

A number of bacterial endotoxins are suggested as excellent tools to study the function of GPCRs, as they covalently modify the α-subunit of G-proteins, altering their function (reviewed in [8]). Pertussis toxin (PT), an exotoxin produced by *Bordetella pertussis* (the causative agent of whooping cough), is shown to be a mono-ADP-ribosyltransferase that covalently modifies the α-subunit of G_i_ proteins. This ribosylation is irreversible and prevents the G-proteins from interacting with G protein-coupled receptors on the cell membrane, thus interfering with intracellular communication [9]–[11]. As a result, the function of effector proteins, such as adenylyl cyclase, ERK/MAPK and Ca^2+^ channels is changed and modulates cell proliferation, survival and angiogenesis [8]. Interestingly, GPCRs antagonists have shown excellent therapeutic benefits in clinical trials in controlling tumor growth and apoptosis [6]. For example, an endothelin A receptor antagonists ZD4054, has been shown to improve the overall survival and reduce the risk of death and bone metastasis in patients with resistant prostate cancer [12]. Therefore, GPCR-based drugs may also show therapeutic benefits in regulation of apoptosis and/or survival in liver diseases.

**Figure 1:**
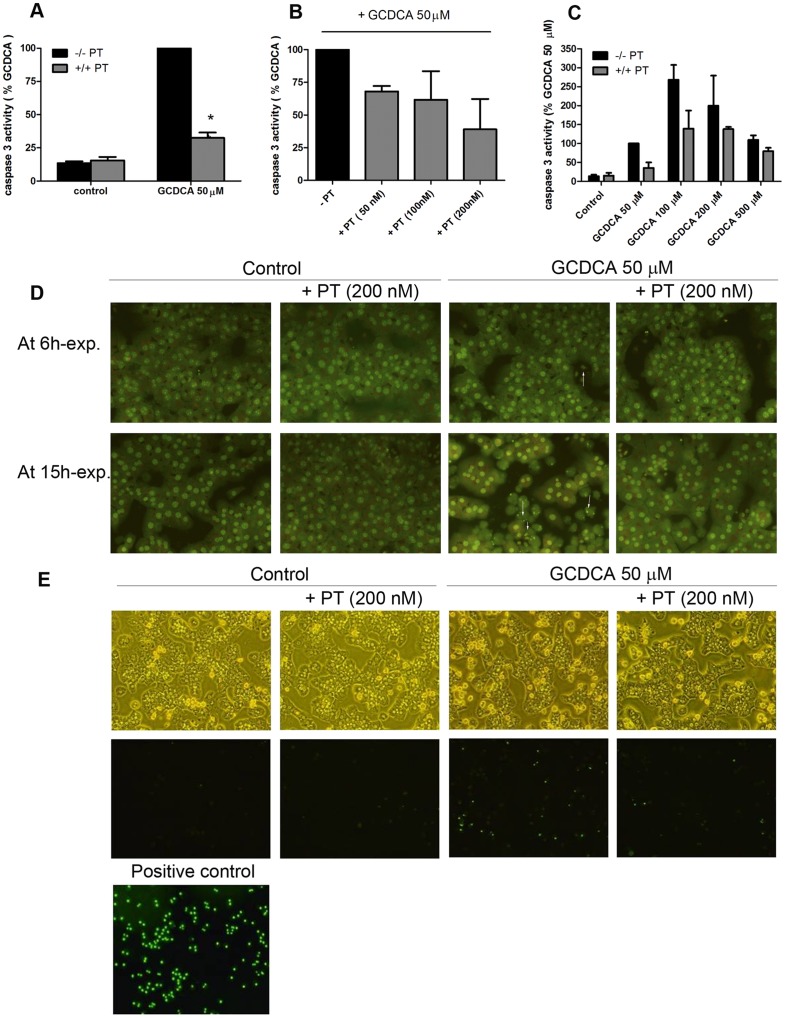
Pertussis toxin (PT) inhibits glycochenodeoxycholic acid (GCDCA)-induced caspase-3 activity and nuclear fragmentation. (**a**) Primary rat hepatocytes were treated for 4 hours with 50 μmol/L of GCDCA, 200 nmol/L of PT or a combination of both. PT was added 30 min prior to the addition of GCDCA. * P<0.05 for GCDCA + PT vs. GCDCA alone. (**b**) Hepatocytes were treated with different concentrations of PT 30 min prior to the addition of 50 μmol/L of GCDCA for 4 hours. (**c**) PT (200 nmol/L) significantly inhibits GCDCA-induced caspase-3 activation. P<0.05 for GCDCA (50, 100, 200 μM) + PT vs GCDCA (50, 100, 200 μM) alone. (**d**) Acridine orange staining. Treatment with 50 μmol/L of GCDCA induces nuclear condensation and fragmentation (white arrows) which is blocked with 200 nmol/L PT at 15 hours after the addition of GCDCA. (**e**) Sytox green staining. PT does not induce necrosis in hepatocytes after 15 hours. Hepatocytes treated with 5 mmol/L H_2_ O_2_ were used as positive control.

**Figure 2:**
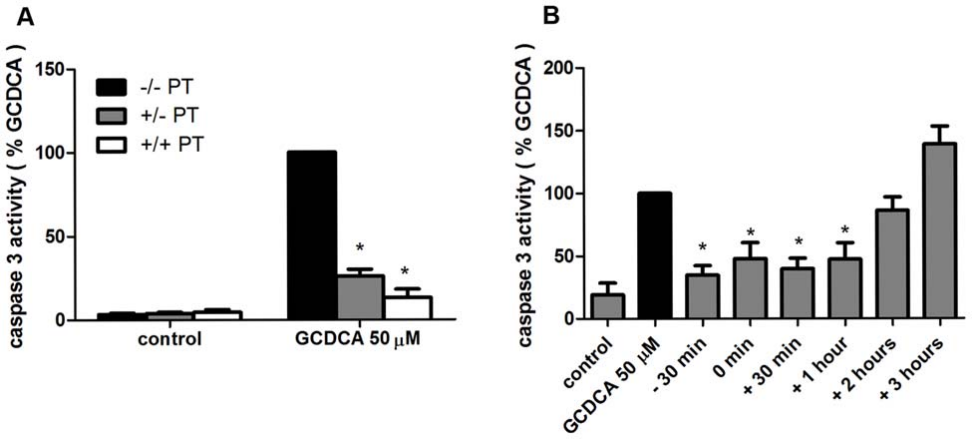
The protective effect of pertussis toxin (PT) against glycochenodeoxycholic acid (GCDCA)-induced apoptosis. (**a**) Hepatocytes were pre-incubated with 200 nmol/L of PT for 15 hours after which cells were washed and exposed to 50 μmol/L of GCDCA alone (+/− PT) or with simultaneous addition of PT (+/+ PT). * P<0.05 for GCDCA + PT (+/−) and GCDCA + PT (+/+) vs. GCDCA 50 μM. (**b**) hepatocytes were stimulated with 50 μmol/L GCDCA for 4 hours (GCDCA 50 µM). PT (200 nmol/L) was added 30 minutes prior to (−30 min), simultaneous with (0 min) or 30 minutes (+30 min), 1 hour (+1 hr), 2 hours (+2 hrs), 3 hours (+3 hrs) after the addition of GCDCA.* P<0.05 for −30 min of PT, 0 min of PT, +30 min of PT, +1 hr of PT vs GCDCA alone.

**Figure 3:**
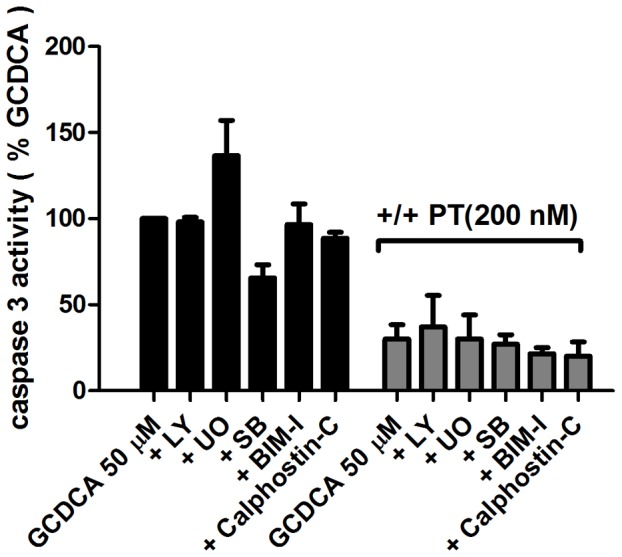
The protective effect of pertussis toxin (PT) is independent of the activation of specific kinases. (**a**) Caspase-3 activity in rat hepatocytes treated with 50 μmol/L of GCDCA in the presence and absence of 200 nmol/L PT and with or without the inhibitors of ERK1/2- MAPK (10 μmol/L of U0126; U0), p38 MAPK (10 μmol/L of SB 203580; SB), PI3K (50 μmol/L of LY 294002; LY), PKC inhibitors (1 μmol/L of calphostin-C, 1 μmol/L of BSM-I).

**Figure 4:**
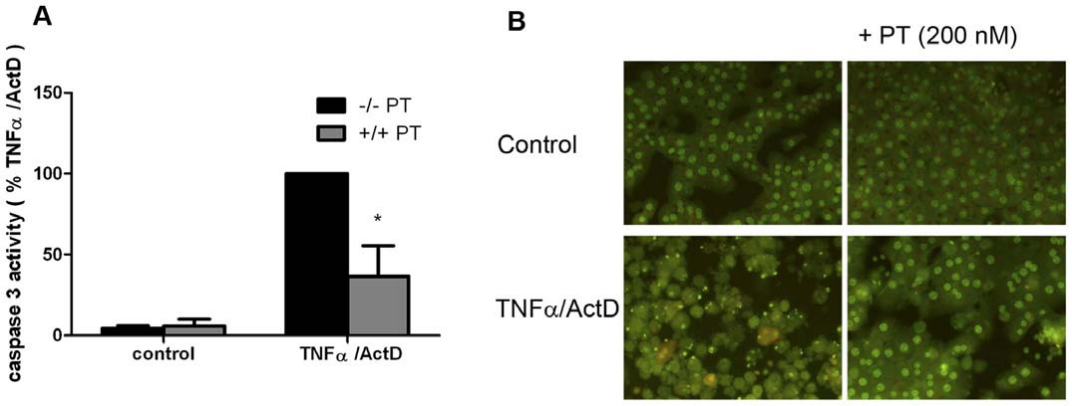
Pertussis toxin (PT) inhibits tumor necrosis factor-α/actinomycin-D (TNFα/ActD)-induced caspase-3 activity and nuclear fragmentation. (**a**) Primary rat hepatocytes were treated for 16 hours with 20 ng/ml of TNFα in the presence of 200 ng/ml of ActD. 200 nmol/L of PT was added 30 min prior to the addition of TNFα/ActD. * P<0.05 for TNFα/ActD + PT vs. TNFα/ActD alone. (**b**) Acridine orange staining. Treatment with TNFα/ActD induces nuclear condensation and fragmentation which is blocked with 200 nmol/L PT.

**Figure 5:**
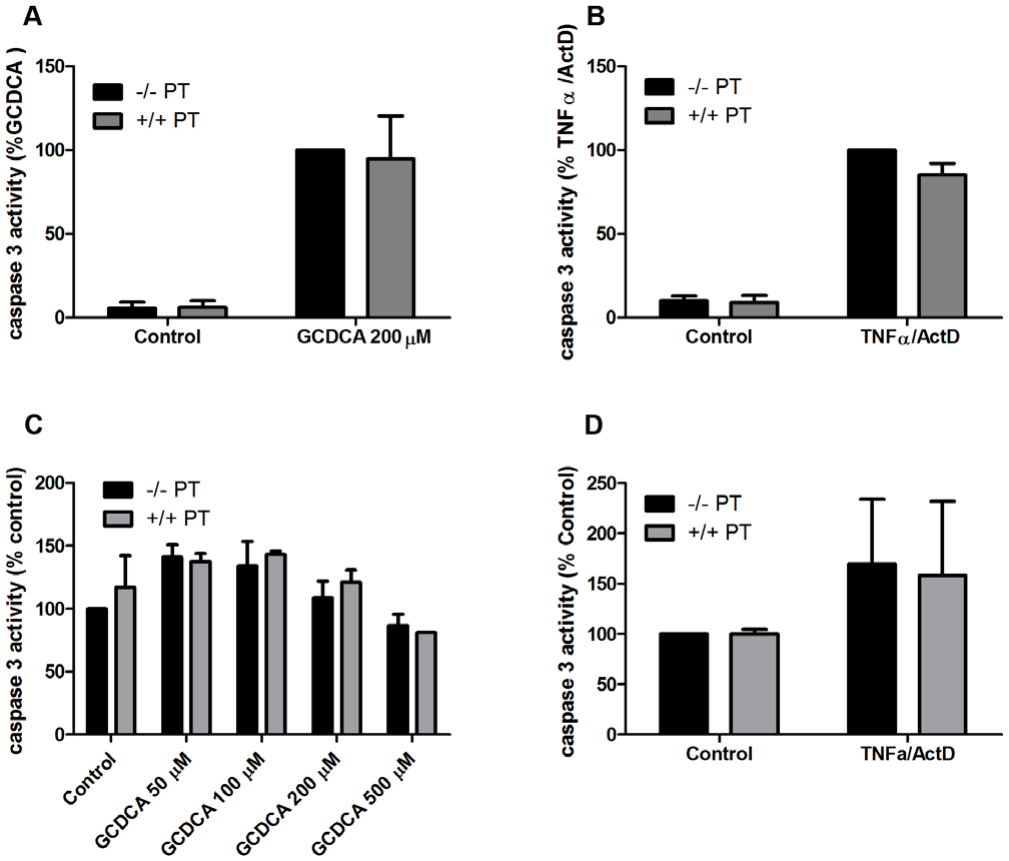
Pertussis toxin (PT) does not inhibit apoptosis in HepG2-rNtcp cells and rat H-4-II-E cells. (**a**) Caspase-3 activity in HepG2-rNtcp cells treated with 200 μmol/L of GCDCA in the presence and absence of 200 nmol/L PT. (**b**) HepG2-rNtcp cells were treated for 16 hours with 20 ng/ml of TNFα in the presence of 200 ng/ml of ActD. 200 nmol/L of PT was added 30 min prior to the addition of TNFα/ActD. (**c**) Caspase-3 activity in rat H-4-II-E cells treated with 50–500 μmol/L of GCDCA in the presence and absence of 200 nmol/L PT. (**d**) Rat H-4-II-E cells were treated for 16 hours with 20 ng/ml of TNFα in the presence of 200 ng/ml of ActD. 200 nmol/L of PT was added 30 min prior to the addition of TNFα/ActD.

GPCRs are present in hepatocytes and play an important role in the regulation of several hepatocyte functions, including gluconeogenesis and lipid storage [13]–[16]. In addition, lysophosphatidylcholine has been shown to act via a G_αi_PCR-dependent mechanism in lipoapoptosis of hepatocytes [15]. Whether PT-sensitive GPCRs also play a role in other apoptotic signals, like bile acid- or cytokine-induced apoptosis, is not known. Liver injury may be caused by (a combination of) inflammation, oxidative stress and increased bile acid levels, leading to hepatocyte cell death. Therefore in this study, we have investigated the effect of PT-mediated inhibition of GPCRs in models of TNFα- and GCDCA-induced apoptosis in primary rat hepatocytes. Our data provide new information about the function of PT-sensitive GPCRs during liver injury and suggest new targets for treatment of liver diseases.

## Materials and Methods

### Animals

Specified pathogen-free male Wistar rats (220–250 g) were purchased from Charles River Laboratories Inc. (Wilmington, MA, USA). Animals were kept under standard laboratory conditions with free access to standard laboratory chow and water. The local Committee for Care and Use of laboratory animals in University of Groningen specifically approved the experiments of this study and all the experiments were performed in accordance with the guidelines of this Committee.

### Rat hepatocyte isolation

Hepatocytes were isolated by a two-step perfusion method using collagenase as described before [17]. In brief, the liver was first perfused through portal vein with Ca^2+^- free Krebs Ringer Hepes buffer, pH 7.4, maintained at 37°C (10 min, flow rate  = 25 ml/min), followed by perfusion with Mg^2+^-free Krebs Ringer Hepes buffer containing Ca^2+^ (5.7 mmol/L) and Collagenase type I (Sigma-Aldrich; 0.12–0.16 U/ml, 10 min, flow rate 8 ml/min). The liver was then removed and placed in the same buffer containing 1% bovine serum albumin (BSA; Sigma-Aldrich) with-out Collagenase. Hepatocytes were then released by gentle teasing of the softened liver and filtered through 60-mesh sterile nylon gauze. The dissected inferior vena cava was used as the outflow port. The buffers were oxygenated prior to perfusion.

Cells were washed three times with HBSS at 50 g for 5 min and the supernatant was discarded. The final cell pellet was re-suspended and cultured in William's E medium in a humidified incubator at 37°C and 5% CO_2_ as described before [18].

### Experimental design

Experiments were started after the attachment period of 4 hours. In order to inhibit the α-subunit of G-proteins, monolayers of cultured primary hepatocytes were treated with pertussis toxin (Calbiochem, VWR International, Amsterdam, the Netherlands) at the indicated concentrations starting 30 minutes prior to the exposure to 50 µmol/L GCDCA (Sigma-Aldrich) for 4 hours or 20 ng/ml recombinant murine TNFα (R&D Systems, Abingdon, United Kingdom) for the indicated time period, unless stated otherwise. Signal transduction pathways were inhibited using 10 µmol/L of the ERK1/2 inhibitor U0126 ( Promega, Madison, USA), 10 µmol/L of the p38 inhibitor SB 203580 (Calbiochem), 50 µmol/L of the PI3 kinase inhibitor LY 294002 (Calbiochem), 1 µmol/L of the protein kinase-C inhibitors Calphostin-C and Bisindolylmaleimide I (BSM-I) (Calbiochem), and 200 ng/ml of the transcriptional inhibitor actinomycin-D (Roche Diagnostics, Almere, the Netherlands). All inhibitors and receptor antagonists were added to the cultured hepatocytes 30 minutes prior to the apoptotic stimuli unless stated otherwise. Each experimental condition was performed in triplicate. Each experiment was repeated at least three times using hepatocytes from different rats. Cells were harvested at the indicated time points as described previously [18].

### HepG2-rNtcp cell experiments

The human hepatoma cell line HepG2 stably expressing the bile acid importer rNtcp was obtained from Dr. Bruno Stieger (Zurich, Switzerland) [19]. The HepG2-rNTCP cells were cultured as described before [20]. Cells were incubated with GCDCA (200 µmol/L) or human recombinant TNFα (20 ng/ml) for the indicated time points. The transcriptional inhibitor actinomycin-D (Roche Diagnostics, Almere, the Netherlands) was added to the cells prior to the addition of TNFα. Cells were treated with pertussis toxin (200 nmol/L, Calbiochem, VWR International, Amsterdam, the Netherlands) 30 minutes prior to the exposure to GCDCA or TNFα/ActD. Cells were harvested at the indicated time points in hypotonic cell lysis buffer [18].

### Rat H-4-II-E hepatoma cell experiments

The rat hepatoma cell line H-4-II-E (European Collection of Cell Culture, Salisbury, UK) was cultured in Earles modified Eagles medium supplemented with 10% FCS, 2 mmol/L glutamine, non-essential amino acids and penicillin/streptomycin/fungizone as described before [21]. Cells were incubated with GCDCA (50–500 µmol/L) or recombinant murine TNFα (20 ng/ml) for the indicated time points. The transcriptional inhibitor actinomycin-D (Roche Diagnostics, Almere, the Netherlands) was added to the cells prior to the addition of TNFα. Cells were treated with pertussis toxin (200 nmol/L, Calbiochem, VWR International, Amsterdam, the Netherlands) 30 minutes prior to the exposure to GCDCA or TNFα/ActD. Cells were harvested at the indicated time points in hypotonic cell lysis buffer [18].

### Apoptosis and necrosis assays

Caspase-3 activity was measured as described previously [18]. The arbitrary fluorescence unit (AFU) was corrected for the amount of protein. Protein concentration was determined using the Bio-Rad protein assay kit. Sytox green (Invitrogen) and acridine orange (Sigma-Aldrich) were used to visualize necrotic and apoptotic cell death, respectively, as described before [22].

### Statistical analysis

Results are presented as the mean of at least 3 independent experiments ± SD. A two-way ANOVA test was used to determine the significance of differences between experimental groups. A P-value of less than 0.05 (P<0.05) was considered statistically significant.

## Results

### Pertussis toxin inhibits bile acid-induced caspase-3 activity and apoptotic nuclear morphology

GCDCA (at 50 µM) induces caspase-3 activity in primary rat hepatocytes that peaks after 4 hours exposure [18]. The effect of PT on GCDCA-induced caspase-3 activity in primary rat hepatocytes was investigated at this time point. Exposure of primary rat hepatocytes to PT (200 nmol/L) alone did not induce caspase-3 activation in hepatocytes (Figure 1a), but significantly inhibited GCDCA-induced caspase-3 activity in a dose-dependent manner, with maximum inhibition observed at 200 nmol/L PT (−60%, P<0.05; Fig. 1a and 1b). In all subsequent experiments a concentration of 200 nmol/L PT was used. PT attenuated the GCDCA-induced caspase-3 activity at different apoptotic concentrations of GCDCA (50–200 µM; Fig. 1c). At higher GCDCA concentrations, the primary mode of cell death shifts to necrosis [22]. PT may delay, rather than prevent GCDCA-induced apoptosis in rat hepatocytes. To establish whether PT prevents, as opposed to delaying apoptosis, GCDCA-treated hepatocytes were stained with acridine orange after 6 and 15 hours exposure with and without PT. Nuclear fragmentation and condensation, markers for end-stage apoptosis, were hardly detectable 6 hours after the addition of GCDCA, but clearly increased over time (shown for 15 hours exposure; Fig. 1d). The formation of fragmented and condensed nuclei was inhibited when GCDCA-exposed hepatocytes (both after 6 and 15 h) were co-treated with PT, comparable to control levels (Fig. 1d). Importantly, PT alone did not induce necrosis in hepatocytes, nor did it increase the number of necrotic cells after exposure to GCDCA (Fig. 1e).

### Time window of anti-apoptotic action of pertussis toxin

PT irreversibly inhibits G_αi_ signaling. If G_αi_ function is required for bile acid-induced apoptosis, pre-incubation with PT alone should also lead to inhibition of GCDCA-induced apoptosis in hepatocytes. Hepatocytes were pre-incubated with PT for 15 hours, which results in complete ADP-ribosylation of Gαi proteins [23]. Medium was removed and cells were washed and then exposed to GCDCA in fresh medium with or without PT for 4 hours. Caspase-3 activity was inhibited in the presence of PT and the protective effect of PT persisted in hepatocytes exposed to GCDCA in fresh medium without PT (Fig. 2a). These data show that the anti-apoptotic effect of PT is sustained in hepatocytes, suggesting that the protective effect of PT is mediated via PT-catalyzed irreversible ribosylation of the α-subunit preventing the G-proteins from interacting with GPCRs [11], [24]. Furthermore, addition of PT up to one hour after the start of the GCDCA treatment still exerted maximum protection against GCDCA-induced apoptosis (Fig. 2b). No protective effect of PT was detected when PT was added at later time points (2–3 h after GCDCA treatment; Fig. 2b). These data indicate that the anti-apoptotic effect of PT is rapidly induced in hepatocytes and suggest the involvement of G_αi_ protein signaling pathways in the protective action of PT against GCDCA-induced apoptosis [23].

### Anti-apoptotic effect of pertussis toxin is not dependent on the activation of ERK1/2, p38 MAP Kinase, PI3-kinase and protein kinase C

To investigate whether specific protein kinase pathways are involved in the anti-apoptotic effects of PT, GCDCA-exposed rat hepatocytes were co-treated with inhibitors of MAPK, PI3K and PKC. These inhibitors alone do not induce caspase-3 activity in rat hepatocytes (data not shown) [18]. The protective effect of PT against GCDCA-induced apoptosis was not abolished by inhibition of ERK1/2, p38 MAP kinases, PI3 kinase and PKC pathways (Fig 3.). These inhibitors at the concentration tested here were effective in our positive control experiments (Data S1 and [18]). This suggests that the anti-apoptotic effect of PT is independent of the activation of these cell survival signaling kinases or that parallel pathways contribute to the protective effect and that inhibition of only one pathway does not result in increased apoptotic cell death.

### Pertussis toxin protects hepatocytes against cytokine-induced caspase-3 activation and apoptotic nuclear morphology

We also analyzed the effect of PT on cytokine-induced apoptosis in hepatocytes. TNFα, in combination with ActD, induces caspase-3 activation in hepatocytes that peaks around 16 hours [25]. PT significantly reduced TNFα/ActD-induced caspase-3 activity in rat hepatocytes (−60%, P<0.05; Fig. 4a). Acridine orange staining confirmed that TNFα/ActD-induced activation of caspase-3 resulted in the formation of fragmented and condensed nuclei, markers of end-stage apoptosis, after 16 hours (Fig. 4b). Importantly, theses markers were absent when TNFα/ActD-exposed hepatocytes were co-treated with PT, confirming that apoptosis was strongly inhibited in the presence of PT (Fig. 4b). Our data show that the PT-sensitive α-subunit of G-proteins is also a key player in TNFα-induced apoptotic signal transduction in primary rat hepatocytes.

### Pertussis toxin does not inhibit apoptosis in HepG2-rNtcp cells and rat H-4-II-E cells

To investigate whether PT has the same anti-apoptotic effect in a human hepatocellular carcinoma cell line and a rat hepatoma cell line as it has in primary rat hepatocytes, we investigated the effect of PT in HepG2-rNtcp cells and rat H-4-II-E cells respectively. HepG2-rNtcp cells and H4-II-E cells were pre-treated with PT for 30 minutes, followed by exposure to GCDCA for 4 hours or TNFα/ActD for 16 hours. PT did not induce caspase-3 activity in HepG2-rNtcp nor did it inhibit GCDCA-induced caspase-3 activation in these cells (Fig. 5a). Similarly, PT did not reduce the TNFα/ActD-induced caspase-3 activity in HepG2-rNtcp cells (Fig. 5b). Although GCDCA and TNFα/ActD did not induce very high levels of active caspase-3 in H-4-II-E (Fig. 5c and Fig. 5d), PT did not increase caspase-3 activity in H-4-II-E cells nor did it inhibit GCDCA-induced caspase-3 activation in these cells (Fig. 5c). Similarly, PT did not reduce the TNFα/ActD-induced caspase-3 activity in H-4-II-E cells (Fig. 5d). These data suggest that the PT-sensitive G_α_-protein is specifically involved in the apoptotic signaling pathways in primary hepatocytes and not in hepatocellular carcinoma cells.

## Discussion

In this study, we report that PT, an inhibitor of G_α_-proteins, protects primary rat hepatocytes against bile acid- and cytokine-induced apoptosis. These effects are specific for primary rat hepatocytes and are not observed in the human hepatocellular carcinoma cell line HepG2-rNtcp or rat hepatoma cell line H-4-II-E cells. We demonstrate that the protective effect of PT is rapidly induced in hepatocytes and is sustained in rat hepatocytes, although the protective action of PT appears to be independent of a single protein kinase (-signaling pathway). We propose that the PT-sensitive α-subunit of G-proteins is a key player in apoptotic signal transduction in rat hepatocytes.

Among the PT-sensitive G-proteins, the G_i_ family is the largest family with wide expression in the different cells and G_βγ_ signaling is usually associated with the G_i_ family [8], [26]. Other PT-sensitive G-proteins have a more restricted distribution in neuroendocrine, visual and lingual tissues [8]. Regulation of gluconeogenesis by bile acids and regulation of lipoapoptosis by lysophosphatidylcholine has previously been shown to be mediated via G_αi_PCR-dependent mechanisms in hepatocytes [13], [15]. Our data suggest that PT-sensitive GPCRs (likely G_αi_PCRs) are involved in bile acid- and cytokine-induced apoptotic signal transduction in primary rat hepatocytes as well.

The specific effect of PT (i.e. catalyzing ribosylation) on the α-subunit of Gi-proteins can explain the rapidly induced and sustained (protective) effect of PT in our experiments. ADP-ribosylated G_αi_-proteins are in an “off” state and cannot transduce GPCR-induced signals [11], [27]. Therefore the receptors that are coupled to these G-proteins are not able to induce signaling pathways upon activation by agonists.

MAP kinase-, PI3 kinase- and PKC-signaling pathways play important roles in regulating hepatocyte death and survival in response to stress inducers [28]–[31]. In addition, the cross-talk between GPCRs and these signaling pathways is responsible for cellular responses, such as cell proliferation, survival, migration and differentiation [32]. Interestingly, our findings indicate that the protective effect of the pertussis toxin against GCDCA-induced apoptosis is not grossly dependent on one of these signaling pathways and may actually be completely independent of these signaling cascades. This finding is remarkable because it has been reported that conjugated bile acids such as GCDCA induce phosphorylation of ERK1/2 and PI3K/Akt pathways in a PT-sensitive manner in primary rat hepatocytes [14]. Thus, one may assume that co-treatment with PT hypersensitizes hepatocytes to GCDCA-induced apoptosis via inhibiting cell survival pathways (e.g., ERK and PI3K/Akt). However, our findings demonstrate the opposite. It is accepted that PT is a general inhibitor for G_αi_-proteins and inhibits several G_αi_PCRs signal transduction pathways [8]. Therefore, it is likely that despite the inhibitory effect of PT on GCDCA-induced ERK1/2 and PI3K/Akt phosphorylation, the overall effect of PT in GCDCA-treated hepatocytes favors cell survival.

TNFα/ActD induces apoptosis in hepatocytes via the activation of death receptor signaling cascade (extrinsic pathway), whereas GCDCA induces apoptosis in hepatocytes via the activation of the mitochondria-dependent apoptotic cascade (intrinsic pathway) [25], [33]. However, there is cross-talk between the bile acid-induced and cytokine-induced signaling cascades in hepatocytes [34], [35]. Ligand-independent transactivation of tyrosine kinase receptors, such as EGFR, is one of the crucial steps in the cross-talk between the intrinsic and the extrinsic apoptotic pathways in rat hepatocytes [35]. Tyrosine kinase receptor transactivation has been associated with GPCRs (reviewed in [36]). Given the involvement of EGFR transactivation in rat hepatocyte apoptosis (Data S2 and [35]) and the anti-apoptotic effect of PT in rat hepatocytes (our data), we suggest that there is cross-talk between PT-sensitive GPCR/G_αi_ and the EGFR, which leads to apoptosis. Indeed, it has been observed that PT pretreatment of hepatocytes inhibited taurodeoxycholic acid (TDCA)-induced activation of the EGFR [14]. It is also reported that PT-sensitive Gi proteins are uniquely involved in the signal transduction pathway mediating EGF-induced activation of phospholipase C-gamma (PLCγ) and Ca^2+^ mobilization (via tyrosine phosphorylation of EGFR) in rat hepatocytes, but not in a rat liver cell line (WB) [37]. Whether GCDCA-induced activation of EGFR in primary hepatocytes is also PT-sensitive remains to be determined.

PT has no effect on apoptotic signaling pathways induced by GCDCA and TNFa/ActD in HepG2-rNtcp cells and rat H-4II-E hepatoma cells. This could be related to differences between (transformed) hepatoma cell lines and primary hepatocytes with regard to gene expression patterns, signaling cascades, GPCRs expression patterns and/or the ability of Gαi proteins to interact with the effector receptors such as EGFR [38], [39]. Indeed, it has been shown that G_αi_ proteins are unable to produce a stable complex with the EGFR or other EGFR-induced signaling protein kinases (such as PLCγ) in the rat epithelial liver cell line, WB [37].

It is known that liver injury is often accompanied by apoptotic hepatocyte cell death [1]. High levels of bile acids, cytokines, reactive oxygen species, drugs and toxins can induce hepatocyte apoptosis. In the liver, massive hepatocyte apoptosis results in acute liver failure, whereas persistent hepatocyte apoptosis and necrosis is often associated with activation of hepatic stellate cells and fibrogenesis, chronic liver dysfunction and end-stage liver disease [1], [3]. Ideally, the most direct therapeutic strategy is to eliminate the cause of the liver injury. However, effective treatments do not exist for many liver diseases, including primary sclerosing cholangitis, NASH, alcohol-mediated hepatitis, viral hepatitis unresponsive to antiviral therapies and acute liver failure. Anti-apoptotic interventions are among the most promising therapeutic strategies for these diseases as they may reduce the apoptosis induced-inflammation and fibrogenesis [1], [40], [41]. GPCRs, are suggested as novel targets for drug innovation, as they have pivotal roles in many physiologic functions (e.g., cell proliferation, angiogenesis, survival) and in multiple diseases including the development of cancer and cancer metastasis. Excellent therapeutic benefits have been observed with some GPCR-based drugs in clinical trials: e.g., the endothelin A receptor antagonists ZD4054 and atrasentan show good antitumor efficacy for ovarian and prostate cancer [5], [6], [42], [43]. GPCRs (e.g., orexin receptor, GPCR78 and LPA receptor) are also involved in the regulation of apoptosis in cancer cells via interaction with different intracellular regulators of apoptosis such as MAPK-, NF-κB- and p53-associated pathways (reviewed in [6]). The participation of GPCR/G_αi_ in hepatocyte apoptosis suggests new targets for drug innovation for (chronic) liver diseases and liver cancer treatment in the future. E.g., activating death receptor-mediated apoptosis in cancer cells by death ligands (such as TRAIL, FasL and TNFα) is suggested as an effective therapeutic strategy in the treatment of hepatocellular carcinoma [44]; however, this strategy may lead to the induction of apoptosis in adjacent normal hepatocytes and loss of functional tissue. In this situation, an adjuvant anti-apoptotic therapy that will inhibit cell death in normal hepatocytes but has no effect on cancer cells may prevent excessive liver damage. Our data suggest that GPCR/G_αi_-based therapeutic strategies may serve as the anti-apoptotic adjuvant therapy, protecting normal tissue against inflammation, bile acid and/or drug-induced cell death.

In summary, our data indicate that GPCRs/G_αi_ participates in several apoptotic signaling pathways in hepatocytes, but not in human hepatocellular carcinoma cells, and that inhibiting the αi subunit of G-proteins is a very effective anti-apoptotic strategy *in vitro.* The participation of GPCR/G_αi_ in hepatocyte apoptosis unveils new targets for drug innovation to treat liver diseases in the future.

## Supporting Information

Data S1 **U0126 (ERK1/2 inhibitor), Calphostin-C and Bisindolylmaleimide I (BSM-I), protein kinase-C inhibitors inhibit ERK and PKC phosphorylation in rat hepatocytes, respectively.** (**a**) Primary rat hepatocytes were treated for 15 min with 20 ng/ml of TNFα in the presence and absence of the inhibitor of ERK1/2- MAPK (10 μmol/L of U0126; U0). Westernblotting was perfomed on cell lysates. Expression of selected protein was assessed using monoclonal mouse antibody against phosphorylated ERK1/2 (p44/42) at a dilution of 1∶1000. Blots were subsequently stripped using 0.1% SDS/0.1% Tween – PBS at 65°C for 30 minutes and incubated with 1∶4000 diluted monoclonal mouse antibody against GAPDH (Calbiochem, La Jolla, CA. USA). Horse radish-peroxidase conjugated rabbit anti-mouse Ig (DAKO, Denmark) was used every time as a secondary antibody at a dilution of 1∶2000. (**b**) Primary rat hepatocytes were treated for 15 min with 50 μmol/L of GCDCA in the presence and absence of the inhibitor of PKC inhibitors (1 μmol/L of calphostin-C, 1 μmol/L of BSM-I). Westernblotting was perfomed on cell lysates. Expression of selected protein was assessed using polyclonal rabbit antibody against phosphorylated PKC (abcam, Cambridge, MA) at a dilution of 1∶500. Blots were subsequently stripped using 0.1% SDS/0.1% Tween – PBS at 65°C for 30 minutes and incubated with 1∶4000 diluted monoclonal mouse antibody against GAPDH (Calbiochem, La Jolla, CA. USA). Horse radish-peroxidase conjugated rabbit anti-mouse Ig (DAKO, Denmark) was used every time as a secondary antibody at a dilution of 1∶2000.
(TIF)

Data S2 **EGFR inhibitor (AG1478) inhibits glycochenodeoxycholic acid (GCDCA)-induced caspase-3 activity in rat hepatocytes.** Primary rat hepatocytes were treated for 4 hours with 50 μmol/L of GCDCA, in the absence or presence of 200 nmol/L of PT and with or without the EGFR inhibitor (25 μmol/L, AG1478). PT and AG1478 were added 30 min prior to the addition of GCDCA. * P<0.05 for GCDCA + PT, GCDCA + AG1478 and GCDCA + PT + AG 1478 vs. GCDCA and alone.
(TIF)
