# Supplementary figures and images for: Pertussis Toxin, an Inhibitor of Gαi PCR, Inhibits Bile Acid- and Cytokine-Induced Apoptosis in Primary Rat Hepatocytes
Source: PLoS One. 2012 Aug 10;7(8):e43156. doi: 10.1371/journal.pone.0043156 (PMC3416748; doi:10.1371/journal.pone.0043156)

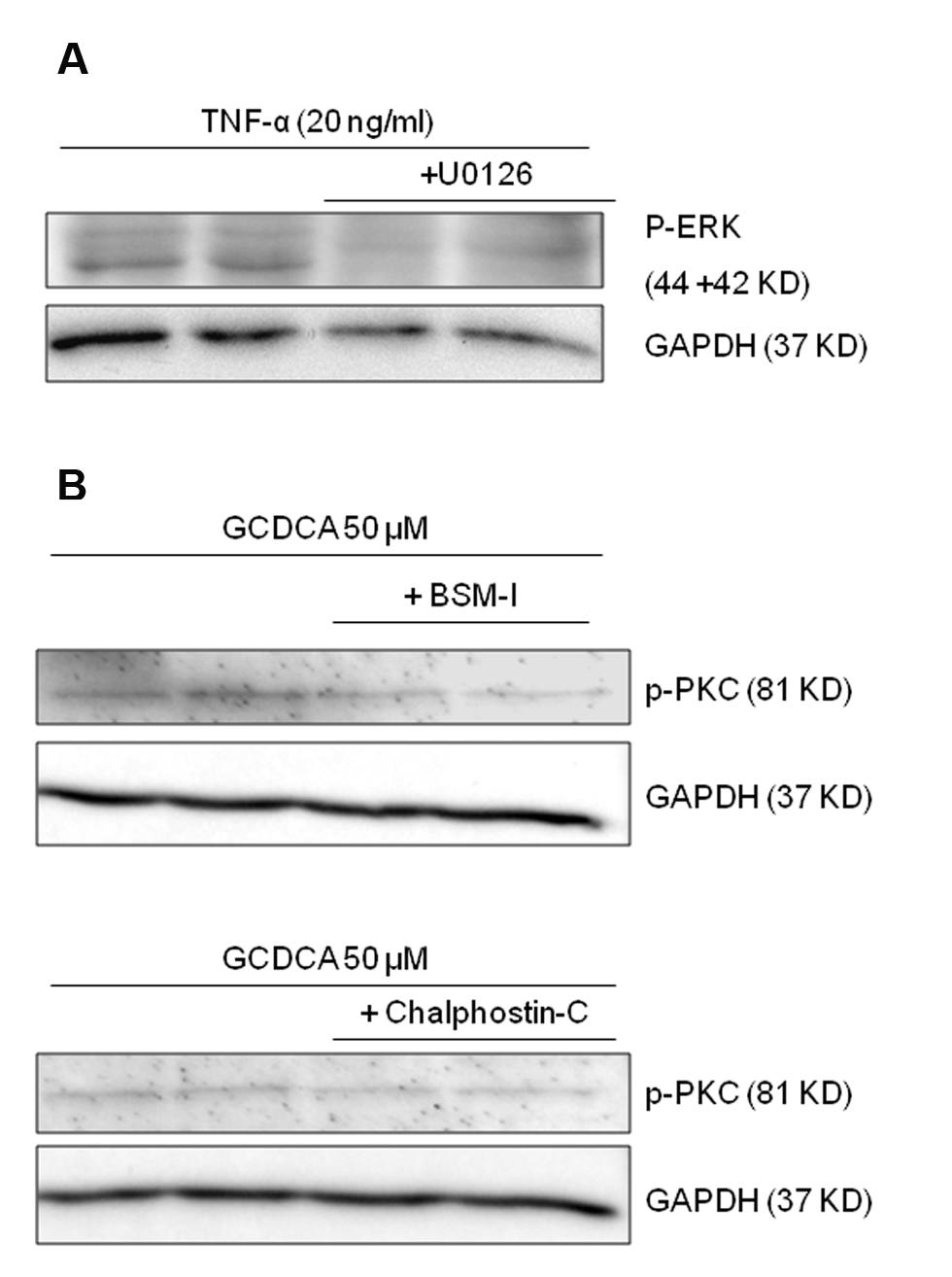

Supplement: Data S1 — U0126 (ERK1/2 inhibitor), Calphostin-C and Bisindolylmaleimide I (BSM-I), protein kinase-C inhibitors inhibit ERK and PKC phosphorylation in rat hepatocytes, respectively. (a) Primary rat hepatocytes were treated for 15 min with 20 ng/ml of TNFα in the presence and absence of the inhibitor of ERK1/2- MAPK (10 μmol/L of U0126; U0). Westernblotting was perfomed on cell lysates. Expression of selected protein was assessed using monoclonal mouse antibody against phosphorylated ERK1/2 (p44/42) at a dilution of 1∶1000. Blots were subsequently stripped using 0.1% SDS/0.1% Tween – PBS at 65°C for 30 minutes and incubated with 1∶4000 diluted monoclonal mouse antibody against GAPDH (Calbiochem, La Jolla, CA. USA). Horse radish-peroxidase conjugated rabbit anti-mouse Ig (DAKO, Denmark) was used every time as a secondary antibody at a dilution of 1∶2000. (b) Primary rat hepatocytes were treated for 15 min with 50 μmol/L of GCDCA in the presence and absence of the inhibitor of PKC inhibitors (1 μmol/L of calphostin-C, 1 μmol/L of BSM-I). Westernblotting was perfomed on cell lysates. Expression of selected protein was assessed using polyclonal rabbit antibody against phosphorylated PKC (abcam, Cambridge, MA) at a dilution of 1∶500. Blots were subsequently stripped using 0.1% SDS/0.1% Tween – PBS at 65°C for 30 minutes and incubated with 1∶4000 diluted monoclonal mouse antibody against GAPDH (Calbiochem, La Jolla, CA. USA). Horse radish-peroxidase conjugated rabbit anti-mouse Ig (DAKO, Denmark) was used every time as a secondary antibody at a dilution of 1∶2000. (TIF) [file pone.0043156.s001.tif]

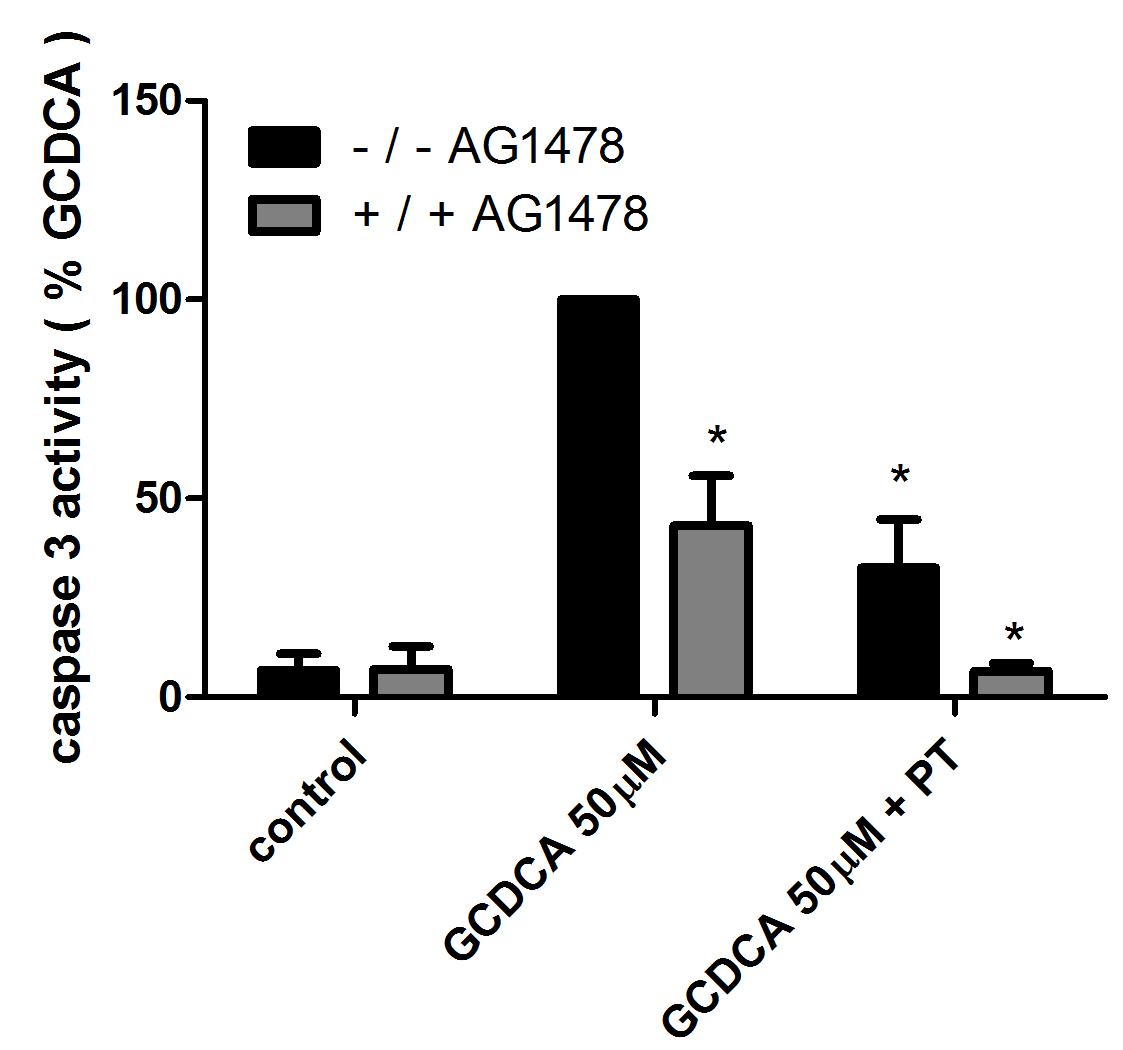

Supplement: Data S2 — EGFR inhibitor (AG1478) inhibits glycochenodeoxycholic acid (GCDCA)-induced caspase-3 activity in rat hepatocytes. Primary rat hepatocytes were treated for 4 hours with 50 μmol/L of GCDCA, in the absence or presence of 200 nmol/L of PT and with or without the EGFR inhibitor (25 μmol/L, AG1478). PT and AG1478 were added 30 min prior to the addition of GCDCA. * P<0.05 for GCDCA + PT, GCDCA + AG1478 and GCDCA + PT + AG 1478 vs. GCDCA and alone. (TIF) [file pone.0043156.s002.tif]
